# Study protocol for a pre-registered randomised open-label trial of ten-session cognitive behaviour therapy (CBT-T) for eating disorders: does stratified augmented treatment lead to better outcomes?

**DOI:** 10.1136/bmjopen-2025-099212

**Published:** 2025-04-25

**Authors:** Tracey Wade, Laura Catherine Edney, Mia L Pellizzer, Jamie-Lee Pennesi, Marcela Radunz, Mike Trott, Yuan Zhou, Glenn Waller

**Affiliations:** 1 Blackbird Initiative, Flinders University Institute for Mental Health and Wellbeing, Flinders University, Adelaide, South Australia, Australia; 2 Queensland Centre for Mental Health Research, Brisbane, Queensland, Australia; 3 Department of Psychology, The University of Sheffield, Sheffield, UK

**Keywords:** Eating disorders, Clinical Trial, MENTAL HEALTH

## Abstract

**Introduction:**

Further improvement of cognitive–behavioural therapy for eating disorders (CBT-ED) is required that can provide better outcomes. Recent work showed that the length of therapy is not critical in improving outcomes. Rather, stratifying the treatment to individual needs is required to produce significant improvements. The current study adopts the approach of evaluating augmentations to ten-session CBT (CBT-T) where initial response to therapy is gradual rather than rapid.

**Methods and analysis:**

Clients aged 15 years and over presenting to the Flinders University Services for Eating Disorders between January 2025 and June 2028 will be randomised to receive either CBT-T as usual or CBT-T augmented with therapy modules (CBT-TA) matched to obstacles to progress for gradual responders. Rapid response, assessed using the Eating Disorder Examination Questionnaire, is defined as ≥1.13 decrease in global ED psychopathology at session 4. In CBT-TA, the therapist and gradual responder will collaboratively choose at least one of nine augmentations to incorporate into therapy. Rapid responders in this group will be given access to the augmentations for use in their own time. Data for the main intent-to-treat analyses will be collected on five occasions: baseline assessment (T1), immediately preceding session 4 (T2), end of treatment (T3) and 3-month and 6-month follow-up (T4 and T5). The primary outcome is ED psychopathology, and secondary outcomes include behavioural indicators of the ED, impairment caused by the ED, general negative emotion, self-harm and hope. Analyses will be undertaken on an intention-to-treat basis and will include all participants in the group to which they were randomised.

**Ethics and dissemination:**

Ethics approval was provided by the Social and Behavioural Research Ethics Committee at Flinders University (7992). This trial was prospectively registered with the Australian New Zealand Clinical Trials Registry (ACTRN12624001495516). The findings arising from the study protocol will be reported to participants and presented at scientific conferences and disseminated by publications submitted to peer-reviewed journals.

**Trial registration number:**

Australian New Zealand Clinical Trials Registry (ACTRN12624001495516).

**STRENGTHS AND LIMITATIONS OF THIS STUDY**

- This parallel two-arm randomised controlled trial examines the benefit of adding augmentation to treatment as usual for non-underweight people with eating disorders.
- This is not an adaptive trial design but will evaluate an adaptive treatment strategy for 10-session cognitive–behavioural therapy (CBT) for eating disorders.
- We will randomise 162 participants to augmented CBT and 68 participants to the non-augmented CBT ensuring sufficient power.
- Gradual responders in the augmented CBT arm will get access to one of nine treatment augmentations designed to tackle obstacles to progress.
- A limitation of the research is that repeated measures assessment of eating disorder psychopathology relies on self-report questionnaires.

## Introduction

 While large within-group effect sizes are obtained for eating disorder (ED) treatments using cognitive–behavioural therapy (CBT) in routine clinical care, with a mean attrition rate of 25.5%,^1^ CBT for bulimia nervosa produces abstinence in only 37.5% of completers.^2^ Further improvement of CBT for EDs (CBT-ED) is required that can provide better outcomes. Recent work shows that the length of therapy is not critical in improving outcomes. A meta-analysis of ten-session CBT (CBT-T) for non-underweight people (ie, excluding anorexia nervosa)^3^ showed pooled within-group effect size (Cohen’s d) reduction in ED psychopathology of −1.49 (−1.68 to −1.31). This is a similar effect size reduction in ED psychopathology achieved by 20-session enhanced CBT, ranging from −1.32 (–1.67 to −0.97)^4^ to 1.79 (−2.21 to −1.37).^5^ CBT-T has five phases, including early dietary change and exposure, behavioural experiments related to food, addressing emotional triggers, body image work and relapse prevention. A key difference from longer forms of CBT-ED is that behavioural and dietary change are stressed from the very outset of treatment rather than being delayed.

Meta-analytic evidence addressing the outcomes of mental health interventions suggests several pathways to producing improvements. First, early change is one of the strongest and most replicable predictors of therapy outcome,^6 7^ with studies of eating disorder treatment measuring this between sessions 3 and 9.^8^ It is also the time when most of the overall change of therapy occurs.^9^,^12^ In CBT-T, two distinctive groups of people receiving therapy can be identified: those who experience ‘rapid response’ and those who experience ‘gradual response’. The former group can be identified as achieving a ≥1.13 decrease in ED psychopathology as measured with the Eating Disorder Examination Questionnaire (EDE-Q),^13^ quantified by Bell and colleagues^14^ using the reliable change index across 164 adults receiving outpatient treatments for EDs. While Bell *et al* considered this change by session 8, subsequent research has shown that it is an effective level of change for identifying early responders at session 4. Application of this decrease by session 4 in a sample of 176 people receiving CBT-T, rapid responders (58% of those commencing treatment) were 2.5 times more likely to meet remission criteria at end of treatment than gradual responders.^15^ At the end of treatment, rapid responders had a significantly lower level of ED psychopathology than gradual responders, with a between-group effect size (Cohen’s d) of −1.11 (−1.50 to −0.72).^15^

Second, stratifying treatment such that it is matched to the individual profile (ie, personalised treatment) improves treatment outcomes. A small but significant effect size favours personalised treatment relative to standardised treatment.^16^ In EDs, the use of stratified treatment for gradual responders has been shown to produce commensurate outcomes between rapid and gradual responders.^17^ Additionally, building in some degree of individual choice and tailoring as to therapy components produces a significant benefit over purely clinician-tailored options.^18^

Third, acute augmentation (defined as interventions delivered immediately before, during or after a session of manualised psychological therapy with the aim of enhancing the impact of the therapy, either as a single intervention or across multiple therapy sessions) of ED treatment has also been shown to significantly improve outcomes over therapy alone with no augmentation.^19^ These acute augmentations are designed to be delivered on at least one occasion and can be repeated but are not offered concurrently over the whole of the treatment duration.

Currently, only five trials in ED exist that use stratified treatment for gradual responders.^15^ To date, none have built in participant choice for personalisation of treatment. This collaborative decision-making is recommended in clinical practice for EDs,^20^ but no rigorous evaluation exists of this practice. We describe a CBT-T treatment protocol designed to reduce the outcome gap between rapid and gradual responders compared with our previous three evaluations of CBT-T.^15^ This augmented treatment, CBT-TA, differs from CBT-T in four main ways: (1) while the first three sessions of the two treatments do not differ, we will now use an a priori indicator based on the global EDE-Q score to differentiate rapid (EDE-Q ≥1.13) and gradual (EDE-Q <1.13) responders before the start of session 4; (2) in the CBT-TA group, gradual responders will have a collaborative discussion with the therapist as to the barriers to more rapid change; (3) these barriers will be matched to nine possible treatment augmentations, identified by a Delphi consensus study across four different panels: people with lived experience, significant others, clinicians and researchers; requiring 80% of participants from all four panels to rate a statement as either ‘Essential’ or ‘Important’ for the statement to be endorsed;^21^ and (4) the gradual responders will receive their choice from nine augmentations, which will be tackled within sessions in addition to treatment as usual over the next five sessions of therapy. In the CBT-TA group, rapid responders will be given access to the platform housing the augmentations, but this content will not be discussed in therapy.

This protocol is reported following Standard Protocol Items: Recommendations for Interventional Trials guidelines.^22^ The overall aim of this randomised controlled trial is to evaluate the comparative effectiveness and acceptability of CBT-T for non-underweight clients with EDs aged 15 years and above to CBT-TA. To achieve this, participants will be randomised to either receive CBT-T or CBT-TA. Our first specific aim is to examine superiority of CBT-TA (in clinically determined gradual responders) against a CBT-T control group. The secondary research aim is to examine superiority of CBT-TA (in clinically determined rapid responders) against a CBT-T control group. We also will address an exploratory aim, namely, whether any differences exist between CBT-TA (passive augmentation) in clinically determined rapid responders vs CBT-TA (active augmentation) clinically determined gradual responders.

First, we hypothesise that gradual responders in CBT-TA will have significantly lower ED psychopathology compared with gradual responders in CBT-T at end of treatment and that these gains will be maintained at the 3-month and 6-month follow-up. We also hypothesise that remission will be higher in the former than latter group. Second, we hypothesise the same advantages for the rapid responder group in CBT-TA over rapid responders in CBT-T. We will also explore (1) change in health service utilisation including primary, secondary and tertiary care, (2) engagement and completion data between the two conditions to establish the relative acceptability of the interventions, and (3) client’s experiences of therapy in the form of qualitative feedback.

## Methods and analysis

### Study design

**Figure 1:**
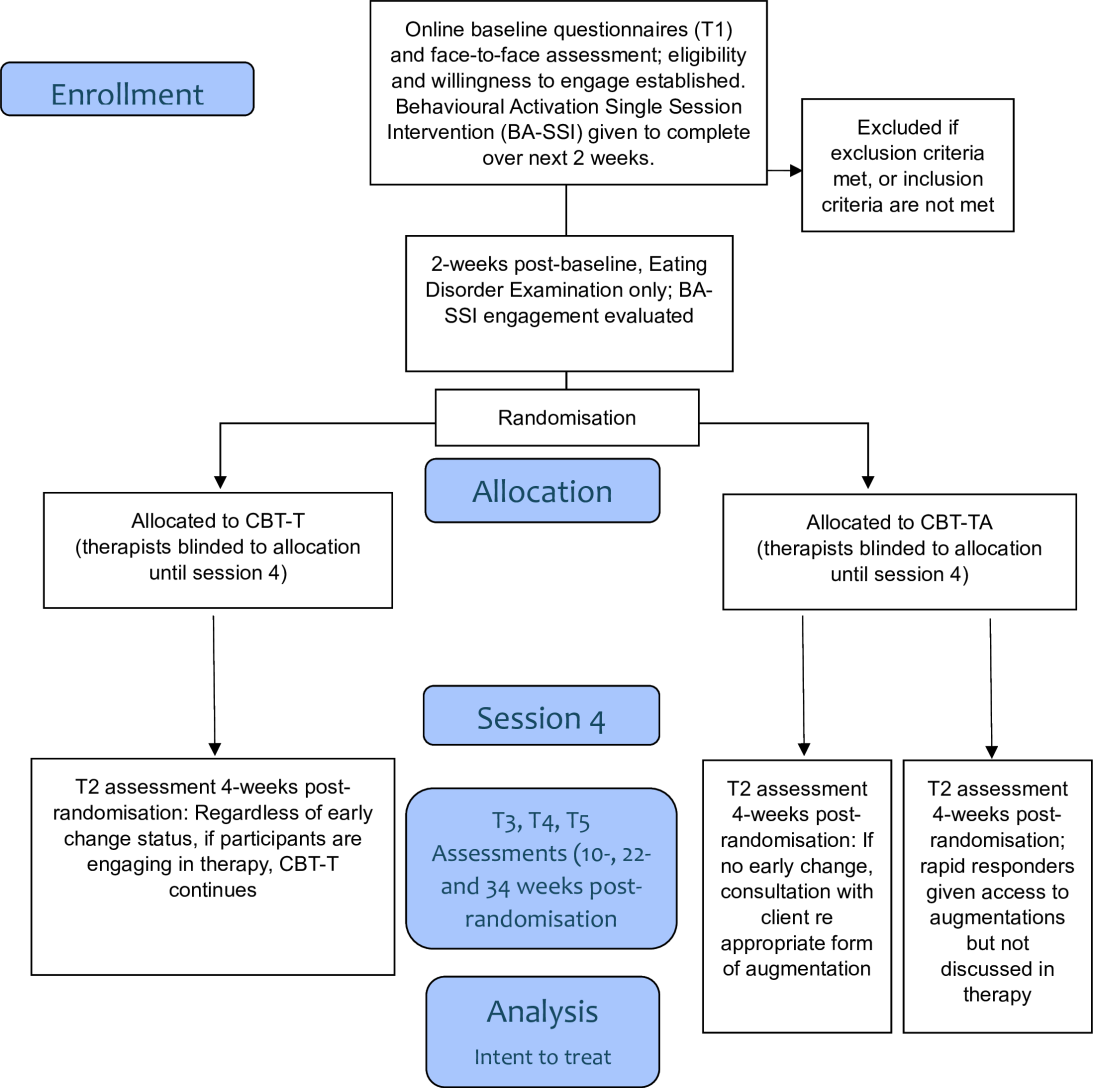
Study design.

The study is a parallel two-arm randomised controlled trial (see figure 1). Demographic data will be collected via the online referral then the full online assessment schedule will occur at baseline assessment (T1) which precedes face-to-face assessment with a therapist to finalise eligibility and desire to engage in CBT-T. Further evaluation of engagement and commitment to therapy will be conducted by asking clients to complete a behavioural activation single session intervention (BA-SSI) over the next 2-week period, at the end of which time (i) ED psychopathology will be assessed online again; (ii) level of engagement with the BA-SSI will be assessed; and (iii) randomisation will occur with the first therapy session occurring within a week. T2 will be conducted immediately preceding session 4, to inform extent of response. Assessments will be repeated at the end of treatment (T3) and 3-month and 6-month follow-up (T4 and T5). T3 will occur 10 weeks post-randomisation (12 weeks post-baseline). T4 and T5 will occur 22 and 34 weeks post-randomisation (24 and 36 weeks post-baseline respectively).

### Study setting

The study will be conducted at the outpatient Flinders University Services for Eating Disorders (FUSED) in South Australia. Previous descriptions of four clinical trials from FUSED have been published,^23^,^26^ each including participants aged 15 years and above. The completer remission rate for CBT-T in our clinic is 36%,^15^ consistent with meta-analytic data for CBT for ED.^2^ In the current study, the CBT-T therapy will be conducted face-to-face by a mix of clinical psychology postgraduate trainees and registered clinical psychologists, under expert supervision. All data are self-reported and will be collected online via the Qualtrics survey platform. Participants can be self-referred or referred by a primary or mental health professional.

### Eligibility criteria

Eligible participants (1) are aged 15 years and over; (2) have any DSM-5 diagnosis of ED as determined by the EDA-5 online clinical interview (https://eda5.org/) excluding anorexia nervosa and avoidant/restrictive feeding intake disorder for which no evidence of the efficacy of CBT-T exists; (3) consent to FUSED communicating regularly with their general practitioner; (4) reside in South Australia; and (5) can read independently at grade 2 level English. The age inclusion was informed by research^27^ showing that adolescents with an eating disorder and a mean age of 15 years self-select CBT rather than family-based treatment. Participants are not eligible if they report current life-threatening suicidal ideation, untreated psychosis, or substance dependence, or have a Body Mass Index (BMI)<18.5 (or BMI-for-age <5th percentile if an adolescent), or currently receiving a psychological therapy for an ED. Eligibility criteria will be assessed at the screening assessment and checked again in the face-to-face assessment.

### Patient and public involvement

The CBT-T protocol^28^ for this study was developed with extensive engagement of clinicians experienced in the delivery of CBT-ED and is associated with a meta-analysis summarising effects across 10 different evaluations.^3^ The focus of the nine therapy augmentations was decided by a Delphi consensus method, conducted online across four different panels: people with lived experience, significant others, clinicians and researchers.^21^ The nine targets or processes include: (1) basing self-worth on one or two aspects of oneself; (2) persistent and excessively high standards; (3) poor distress tolerance skills and emotion regulation problems; (4) being self-critical; (5) negative body image; (6) low self-compassion; (7) low self-worth and self-acceptance; (8) social isolation; and (9) unhelpful thinking habits. The augmentations will be interventions that have robust evidence for ability to improve these processes (eg, behavioural activation for emotional regulation or social isolation) delivered via online interactive documents that have been co-designed with our expert advisory panel, consisting of people with lived experience, carers, researchers and clinicians.

### Interventions

The CBT-T protocol^28^ forms the basis of both intervention arms. The assessment period for both the CBT-T and CBT-TA arms is designed to encourage change and treatment retention and improve treatment outcome before treatment commences. First, the face-to-face assessment will include motivational enhancement exercises that have been shown to significantly improve outcomes for less motivated clients.^25^ Second, completion of an SSI between the assessment and start of therapy has been shown to significantly increase completion of the 10 therapy sessions.^26^ Third, our current, unpublished research shows that use of the BA-SSI (as an interactive document available at https://osf.io/xpqa8/) in the 2-week period between assessment and treatment significantly reduces ED psychopathology in 69% of participants. This suggests that early change sets them up for better outcomes in treatment.

The intervention comprises of 10 face-to-face sessions with each session lasting approximately 50 min. The CBT-T protocol which describes the structure of each session in detail is available on the CBT-T website: https://cbt-t.sites.sheffield.ac.uk/resources. A further three sessions will be scheduled after treatment ceases to ensure that progress is maintained (1, 3 and 6 months post-treatment); assessment will only be conducted at 3-month and 6-month follow-up (T4, T5).

In CBT-T, a review of engagement and progress is routinely conducted in session 4, where those who are not engaging in therapy (eg, are not actively doing the therapy homework tasks every day between sessions, such as food monitoring, exposure tasks) are invited to step away from treatment until circumstances allow them to fully commit to doing therapy, or to have one final attempt to engage by session 5. In the current study, progress will be examined using sessional assessment with the ED15.^29^ Rapid response will be classified as a ≥1.13 decrease in global ED psychopathology by session 4 as measured with the EDE-Q.^13^

Novel to this study, CBT-TA will give participants access to nine treatment augmentations in the form of an interactive document. The gradual responders will be engaged in discussion at session 4 to examine barriers to rapid change and which of the augmentations may best address these. The augmentations will then be incorporated into ongoing therapy. The rapid responders will be made aware of the treatment augmentations and how to locate them, but they will not be incorporated into ongoing therapy.

### Procedure and participant Timeline

Figure 1 outlines the study flow and table 1 outlines the schedule of enrolments, interventions and assessments. People can self-refer by contacting the FUSED email (fused@flinders.edu.au) or be referred by a primary health (eg, general practitioner) or mental health professional. Tracey Wade’s Flinders University website contains a link (https://www.flinders.edu.au/engage/community/clinics/flinders-university-services-eating-disorders) to information about the study. People wishing to proceed to a face-to-face assessment will either, depending on therapist availability, (**i**) be placed on a waitlist or (ii) be emailed with a date for an assessment appointment and a Qualtrics link to the T1 assessment and online consent, to be completed before the appointment. People under the age of 18 years require parental consent as well as their personal assent. The study and the requirements of therapy will be explained in the face-to-face assessment, and participants who are interested in doing therapy will be asked to sign consent to allow FUSED to contact their general practitioner and other professionals involved in their care.

**Table 1:** Schedule of assessment

Timepoint	Initial assessment	Pre-treatment				
	T_1_		T_2–S4_	T_3–S10_	T_4–FU1_	T_5–FU2_
Enrolment:
Eligibility screen	X
Informed consent	X
Given BA-SSI to complete	X
Allocation	X
Interventions:
CBT-T	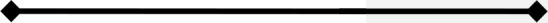			
CBT-TA	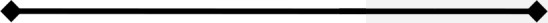			
Assessments:
Eating Disorder Examination-Questionnaire	X	X	X	X	X	X
Service use	X	X
General negative emotion (DASS)	X	X	X	X	X
Inventory of Statements About Self-Injury (ISAS) Section I: Behaviours	X	X	X	X	X
(ISAS) Section II: Functions	X
Clinical Impairment Questionnaire	X	X	X	X	X
Adult State Hope Scale	X	X	X	X	X
BA-SSI completion	X
Qualitative feedback: worst and best things about therapy	X
ED15 (used before each session)	X	X	X	X	X

BA-SSI, behavioural activation single session intervention; CBT-T, ten-session cognitive–behavioural therapy.

Following the baseline assessment, participants are given the BA-SSI (either in hard copy or emailed as an interactive document) and an appointment for a first session of therapy will be made for 2 weeks post-assessment. A follow-up email will be sent to the participant containing the Qualtrics link for the T2 assessment, to be completed before the first session of therapy. If this is not completed by the morning of the scheduled appointment, the therapy will not proceed, and the participant will be contacted as to whether they wish to pursue therapy. If they do not proceed into the study, a letter will be sent to them and shared with their general practitioner, summarising the assessment and treatment needs, and alternative services.

Once the participant attends the first therapy appointment, randomisation of the participant to either the CBT-T or CBT-TA condition will be recorded. The therapist and client will be blind to this allocation and will be informed of randomisation between sessions 3 and 4 of therapy.

### Primary outcomes

The primary outcome is ED psychopathology, measured using the global score from the EDE-Q^13^ over the past 28 days. The global score can range from 0 to 6, and higher scores indicate greater psychopathology. This measure has adequate psychometric properties^30 31^ and is widely used to assess and monitor EDs in clinical practice and treatment outcome studies.

### Secondary outcomes

The secondary outcomes include behavioural indicators of the ED, impairment caused by the ED, general negative emotion, self-harm and hope.

We will examine the five behavioural items on the ED15, used at each session. The ED15 is a 15-item questionnaire that assesses behaviours and cognitions during the previous week.^29^ Six items assess weight and shape concerns (eg, “felt distressed about my body shape”) and four assess eating concerns (eg, “worried about losing control over eating”) on a seven-point Likert scale (0=not at all to 6=all the time). Higher ratings indicate higher levels of psychopathology. Five additional questions assess the frequency of disordered eating behaviours (binge eating, vomiting, laxatives, excessive exercise, restriction), but these do not contribute to those two scales. The reliability and validity of the ED15 have been supported.^32^

The 16-item Clinical Impairment Assessment Questionnaire (CIA)^33^ is a self-report measure of psychosocial impairment in the past 28 days attributed to experiencing an ED. Impact on areas of functioning such as mood and self-perception, cognitive functioning, work performance and interpersonal functioning is measured on a 4-point Likert scale (0=not at all to 3=a lot). The CIA correlates with ED psychopathology and has good discriminant^34^ and predictive validity.^35^

The total score of the 21-item version of the Depression Anxiety Stress Scale (DASS-21)^36^ will be used, where factor analysis indicates that a general factor of psychological distress or general negative emotion exists.^37^ Participants rate the extent to which a statement applies to them in the past week on a 4-point Likert scale (0=never, 3=almost always). Higher scores indicate higher severity of symptoms. The DASS-21 has sound psychometric properties, including internal consistency, convergent validity and discriminant validity.^38^

Section I of the Inventory of Statements About Self-Injury (ISAS)^39^ assessing behavioural items will be used to measure self-harm. The ISAS has been shown to have satisfactory psychometric properties, including test-retest reliability.^39 40^ Seven items assess type and frequency of self-harm behaviours.

The 8-item Adult Hope Scale^41^ will be used to measure dispositional hope. Each item is scored on an 8-point Likert scale, ranging from ‘definitely false’ to ‘definitely true’. Higher scores represent higher levels of hope. As well as a total score, two subscales can be identified that measure pathway thinking (eg, I can think of many ways to get out of a jam) and agency thinking (eg, I energetically pursue my goals). The scale has been found to be valid and reliable in adults.^42^

### Other measures

Other measures include demographics, functions of self-harm, healthcare use, and feedback about the service (aspects least and most liked), and acceptability as indicated by the number of sessions completed and premature cessation of therapy (drop-out). Demographic information will include ED diagnosis, age, duration of the ED, cultural identity, previous mental health treatment, gender, socioeconomic status, and one item assessing importance of change and ability to change. The functions of self-harm will be assessed only at baseline to inform treatment, using Section II of the ISAS,^43^ consisting of 39 items scored on a 3-point scale: not relevant, somewhat relevant and very relevant. There are 13 function subscales: affect regulation, interpersonal boundaries, self-punishment, self-care, anti-dissociation/feeling-generation, anti-suicide, sensation-seeking, peer-bonding, interpersonal influence, toughness, marking distress, revenge and autonomy. Healthcare use will assess the type and frequency of healthcare use over the previous 3-month period. The BA-SSI completion item will ask participants to estimate how much of the SSI they completed, on a sliding scale from 0 to 100.

### Sample size

A previous randomised controlled trial completed at FUSED showed that less motivated clients receiving CBT augmented with a small amount of motivational content had significantly better outcomes at 1-month follow-up than those who did not receive this content.^25^ At 3-month follow-up, a between-group effect size (Cohen’s d) benefit persisted of 0.37 (−0.20 to 0.93). Based on a power of 0.80, alpha of 0.05, assuming a correlation of 0.50 between baseline and post-training assessments, a small between-group effect size d=0.35 and 10% attrition between each of the 5 waves of data collection (40% attrition in total), we will be required to randomise 162 participants to CBT-TA and 68 participants to CBT-T (n=230).^44^ Over previous trials, around 50 clients have completed treatment at FUSED annually. To ensure we can randomise 230 clients over 4 years, we will open referrals to people with binge eating disorder (previously not eligible for a service), add four registered clinical psychologists who will have an ongoing client load of 2–5 people to the usual staffing of clinical psychology postgraduate trainees, and advertise the service.

### Recruitment

Participants will be recruited in South Australia between January 2025 and June 2028. Information about the study will be distributed to headspace centres (primary mental health services for youth), the Statewide Eating Disorder Service, and the Primary Healthcare Network that administers the National Eating Disorder Collaboration funded ‘Right Care Right Place’ co-ordination of ED referrals to Head to Health sites.

### Randomisation and blinding

A non-balanced randomisation ratio of 2.4:1 (CBT-TA:CBT-T) will be used to account for 42% of participants being eligible for the final analysis related to our primary aim that is, based on a previous evaluation finding 58% of FUSED clients to meet criteria for rapid response.^15^ Random allocation will occur after the first intervention session using an Excel sheet generated by Sealed Envelope (https://www.sealedenvelope.com/) before the trial commenced. Participants and therapists will be blinded up to session 4. The research team will not be blinded to allow for monitoring and contact with participants, but evaluation of treatment effects on primary and secondary outcomes post-training will be conducted by a researcher (LE) blinded to treatment condition.

### Data collection, management and statistical analysis

Participant data will be collected on Qualtrics and securely stored on the Flinders University R drive and accessible only by the Flinders University research team. Participant names will be used to link surveys across assessment points and to usage data. Participant names will be deleted when data is downloaded from Qualtrics for analysis purposes and each participant will be assigned a randomly created user ID for deidentification purposes.

Interim analyses will be conducted to monitor the ratio of rapid responders vs gradual responders when 33% (n=48 in the active arm) and 66% (n=96 in the active arm) participants have completed session 4 (and therefore, have been categorised as rapid or gradual responders). Results from interim analyses will inform future randomisation ratios, with randomisation ratios being adapted if the proportion of rapid responders is ±10% of the assumed response rate of 58%. Results of interim analyses will be non-binding and subject to trial management group approval.

Analyses will be undertaken with a treatment-policy approach (ie, intention-to-treat) and will include all participants in the group to which they were randomised, regardless of actual receipt or uptake of the intervention or withdrawal from the study (ICH E9 R1^45^). Mixed-model repeated measures analysis will be used for the continuously scaled primary outcome and secondary outcome variables. An unstructured residual variance-covariance matrix will accommodate within-participant dependency. The model will include factors of study condition (CBT-T, CBT-TA), occasion of measurement (baseline, 4, 10, 22 and 34 weeks post-randomisation) and their interaction as fixed effects, with a random intercept to account for variation between individual participants. The primary outcome will be assessed by a planned comparison of the difference between groups in change of the primary outcome variables over time. Given the assumption of MRMM that data are missing at random (MAR), multiple imputation will be used to replace missing observations. To examine robustness of the results and the MAR assumption, sensitivity analyses will be conducted using the tipping point and delta adjustment method. Additionally, we will run a number needed to treat analysis, based on the proportion of participants in each arm who meet the prespecified criteria for remission,^46^ (i) global EDE-Q score ≤2.77, (ii) BMI ≥18.5 and (iii) no ED behaviours in the previous 1 month period.

Analyses of secondary outcome variables will follow the same methods as the primary outcome except for the behavioural count variables, which will be analysed with generalised linear mixed modelling (GLMM). Separate GLMM models with a log link function and gamma distribution will be used to account for the right skewed health utilisation data.

### Monitoring

The trial is overseen by the principal investigator (TDW) and the trial management group (Pellizzer, Pennesi, Radunz, Zhou). Day-to-day trial oversight will be provided by a PhD student (Raima Harding) who will meet with the principal investigator on a weekly basis. All adverse events and serious adverse events and broader safety monitoring will be documented and reported to the trial management group by the principal investigator and reported in the primary outcomes paper. If there are concerns for participant safety, based on (but not limited to) deterioration of mental health, the trial management group may recommend pausing or terminating the trial. Adverse events will be reported to the ethics committee if appropriate. The trial is insured with AON policy # AUS21899001.

## Ethics and dissemination

Flinders University is the sponsor of this clinical trial and ethics approval was provided by the Social and Behavioural Research Ethics Committee at Flinders University (7992). The information and consent form for adults and minors can be found in onlinesupplements 1  2 respectively. The trial is covered by Flinders University general and liability insurance protections that will indemnify Flinders University staff and students. This trial was prospectively registered with the Australian New Zealand Clinical Trials Registry (20 December 2024; ACTRN12624001495516). The findings arising from the study protocol will be reported to participants and presented at scientific conferences and disseminated by publications submitted to peer-reviewed journals. The deidentified data set used and/or analyses will be available from the corresponding author’s Open Science Framework site.

## Supplementary material

10.1136/bmjopen-2025-099212 online supplemental file 1

10.1136/bmjopen-2025-099212 online supplemental file 2

## Data Availability

No data are available.
