# Supplementary material for: Study protocol for a pre-registered randomised open-label trial of ten-session cognitive behaviour therapy (CBT-T) for eating disorders: does stratified augmented treatment lead to better outcomes?
Source: BMJ Open. 2025 Apr 25;15(4):e099212. doi: 10.1136/bmjopen-2025-099212 (PMC12035431; doi:10.1136/bmjopen-2025-099212)
Supplement: online supplemental file 1 [file bmjopen-15-4-s001.pdf]

Flinders University

PARTICIPANT INFORMATION SHEET AND CONSENT FORM

Title: A randomized trial of ten-session cognitive behaviour therapy (CBT-T) for eating disorders

Chief Investigator:
Matthew Flinders Distinguished Professor Tracey Wade
College of Education, Psychology and Social Work, Flinders University
Email: tracey.wade@flinders.edu.au

Co-Investigators (from the College of Education, Psychology and Social Work, Flinders University):
Dr Mia Pellizzer
Email: mia.pellizzer@flinders.edu.au

Dr Yuan Zhou
Email: joanne.zhou@flinders.edu.au

Dr Jamie-Lee Pennesi
Email: Jamie-lee.pennesi@flinders.edu.au

Dr Marcela Radunz
Email: marcela.radunz@flinders.edu.au

Dr Laura Edney
Email: laura.edney@flinders.edu.au

Ms Raima Harding
Email: raima.harding@flinders.edu.au

Professor Glenn Waller
Department of Psychology
Sheffield University, United Kingdom
Email: g.waller@sheffield.ac.uk

Description of the Study

We are evaluating the best way to provide ten-session cognitive behaviour therapy (CBT-T) to people with eating disorders. CBT-T is provided to you by postgraduate clinical psychology students or registered psychologists under expert supervision.

Purpose of the study

The aim of this study is to see if we can help people with eating disorders get more out of therapy by supporting them with useful information before they start therapy and during the middle of therapy if there has been a gradual rather than fast response to treatment.

Benefits of the study?

We cannot guarantee or promise that you will receive any benefits from this research; however, our previous evaluations of this therapy show that people have reported substantial improvements in their eating disorder and quality of life.

Participant involvement and potential risks

If you agree to participate in the research study, you will be asked to:

- Attend an initial assessment at the Flinders University Service for Eating Disorders (FUSED) to discuss whether this study would suit you - you must be at least 15 years old, have a body mass index of 18.5 or above, have an eating disorder, and be willing for the therapist to communicate with your general practitioner. You won't be able to do the treatment if you have a severe psychiatric condition that would interfere with treatment (e.g., high suicidality, active psychosis), if you are already receiving therapy for an eating disorder, if you have difficulty speaking or understanding English.
- Decide if the treatment would suit you, and if so, you will start the treatment 2 weeks after the initial assessment. During this time, you will complete a short intervention (50 minutes) to help you think about how you can increase pleasurable activity in your life and try to provide your body with the nutrition it needs for participation in life.
- Treatment involves 10 sessions of face-to-face CBT held in a private therapy room at FUSED, provided by post-graduate trainee psychologists supervised by Professor Wade and Dr Pellizzer, or a registered psychologist or clinical psychologist (the therapist type is dependent upon availability). There are no costs associated with participating in this research and you will not be paid. If at any point you decide that you do not want to receive the CBT, this is not a problem. Sometimes you need to try different approaches before you find the one that works for you. We will write to you and your GP to provide a list of alternative options. Throughout the study, you can request any support person to be present, and you can also ask for special additional sessions for a significant other and yourself.
- Before starting treatment, you will be randomly assigned to one of two different groups. Both groups will receive CBT-T. At session 4 of treatment a review occurs. In one group, you will continue treatment if you are engaging (fully participating) in the treatment. For the second group, if you and your therapist feel there has not been early change in your symptoms, you will decide together on what needs to be added to treatment. This is at least one extra, small (up to 50 minutes) treatment that you can complete in one of the following areas: (1) basing self-worth on one or two aspects of oneself; (2) persistent and excessively high standards; (3) poor distress tolerance skills and emotion regulation problems; (4) being self-critical; (5) negative body image; (6) low self-compassion; (7) low self-worth and self-acceptance; (8) social isolation, (9) unhelpful thinking habits. If there has been early change in your symptoms, and you are in this second group, you will have access to these extra treatments too.
- The treatment review that occurs in session 4 will be video-taped to help us understand how this decision is made and so we can continue to improve the effectiveness of this therapy. Complete questionnaires on six occasions over a 24-week period up to 30 minutes on each occasion, a total of 3 hours of assessment. Data will be collected by your therapist by sending you a link to a questionnaires which will be completed online and include questions/statements relating to disordered eating, self-harm, cognitive impairment, hope, depression, anxiety, and stress. We ask about sensitive issues such as self-harm, whether life is meaningless, and feeling disgust about one-self. We will also ask about your expectations for treatment, demographic information, healthcare use, motivation and ability to change, and feedback about the treatment and service. The assessments occur before your initial assessment, after 2 weeks, then 4-weeks after that, at the end of therapy, and then 12 and 24 weeks after therapy has finished.

There are risks with undertaking any psychological treatment, and the questionnaires and CBT deal with some sensitive personal issues. If you need, you may also contact the following free services:

- Flinders University Health, Counselling, and Disability Services (Level 3, Student Services Centre, open 8.45am to 5pm Monday to Friday) on (08) 8201 2118
- Lifeline on 13 11 14,
- Statewide Eating Disorder Service (open 9am to 4.30pm Monday to Friday) on (08) 8198 0800,
- Beyond Blue (open 24/7) on 1300 224 636,
- Suicide Call Back on 1300 659 467
- Butterfly National Helpline for eating disorders (available 8am to midnight, 7 days a week) on 1800 33 4673.

Withdrawal rights
You may decline to take part in this research study. If you decide to take part and later change your mind, you may, withdraw at any time without providing an explanation. Let your therapist know if you want any data collected up to the point of your withdrawal to be securely destroyed. You will be offered a debriefing session with your therapist to plan further care.

Confidentiality and Privacy
Only researchers listed on this form have access to the individual information provided by you. Privacy and confidentiality will be always assured. The treatment review in session 4 will be video recorded and transcribed using Microsoft Teams in-built transcription software which will be checked manually by the researchers and de-identified. The research outcomes may be presented at conferences, written up for publication or used for other research purposes as described in this information form. However, the privacy and confidentiality of individuals will be always protected. You will not be named, and your individual information will not be identifiable in any research products. No data, including identifiable, non-identifiable and de-identified datasets, will be shared or used in future research projects without your explicit consent.

Data Storage
The information collected will be stored securely on a password protected computer and Flinders University server for 7 years. Any identifiable data will be de-identified for data storage purposes. With your consent, these data will also made available permanently on the Open Science Framework where other researchers around the world can request permission to analyse the data for the purposes of replicability or meta-analysis.

Recognition of Contribution / Time / Travel costs
There is no reimbursement associated with participating in this research.

How will I receive feedback?
Following project completion, a brief report of the outcomes of the project will be provided on Professor Tracey Wade's web page and can also be emailed to you on request.

Ethics Committee Approval
The project has been approved by Flinders University's Human Research Ethics Committee (*7992).*

Queries and Concerns
Queries or concerns regarding the research can be directed to the research team. If you have any complaints or reservations about the ethical conduct of this study, you may contact the Flinders University's Research Ethics & Compliance Office team via telephone 08 8201 2543 or email human.researchethics@flinders.edu.au.

Thank you for taking the time to read this information sheet which is yours to keep. If you accept our invitation to be involved, please complete the following Consent Form.

Flinders University

CONSENT FORM

Consent Statement

☐ I have read and understood the information about the research, and I understand I am being asked to provide informed consent to participate in this research study. I understand that I can contact the research team if I have further questions about this research study.

☐ I am not aware of any condition that would prevent my participation, and I agree to participate in this project.

☐ I understand that I am free to withdraw at any time during the study.

☐ I understand that I can contact Flinders University's Research Ethics & Compliance Office if I have any complaints or reservations about the ethical conduct of this study.

☐ I understand that my involvement is confidential, and that the information collected will be published. I understand that I will not be identified in any research products.

I further consent to:

☐ completing questionnaires
☐ having my treatment review conversation in session 4 video taped
☐ sharing my de-identified data with other researchers on the Open Science Framework
☐ my de-identified data being used in this project and other related projects for an extended period of time

Signed:

Name:

Date:
