# Supplementary material for: Study protocol for a pre-registered randomised open-label trial of ten-session cognitive behaviour therapy (CBT-T) for eating disorders: does stratified augmented treatment lead to better outcomes?
Source: BMJ Open. 2025 Apr 25;15(4):e099212. doi: 10.1136/bmjopen-2025-099212 (PMC12035431; doi:10.1136/bmjopen-2025-099212)
Supplement: Supplementary file 3 [file bmjopen-2025-099212.reviewer_comments.pdf]

PEER REVIEW HISTORY

BMJ Open publishes all reviews undertaken for accepted manuscripts. Reviewers are asked to complete a checklist review form and are provided with free text boxes to elaborate on their assessment. These free text comments are reproduced below.

ARTICLE DETAILS

Title (Provisional)

Study protocol for a pre-registered randomized open-label trial of ten-session cognitive behaviour therapy (CBT-T) for eating disorders: Does stratified augmented treatment lead to better outcomes?

Authors

Wade, Tracey; Edney, Laura Catherine; Pellizzer, Mia L; Pennesi, Jamie-Lee; Radunz, Marcela; Trott, Mike; Zhou, Yuan; Waller, Glenn

---

VERSION 1 - REVIEW

**Reviewer**	**1**
**Name**	**Goel , Neha J.**
**Affiliation**	**Virginia Commonwealth University**
**Date**	**08-Feb-2025**
**COI**	**None**

Summary: This manuscript reports on a protocol for a randomized clinical trial of CBT-TA for rapid and gradual responders compared to CBT-T. I enjoyed reading this protocol and look forward to reviewing study findings in the coming years. Please see below for my general recommendations.

Major Revisions:

1. Please modify the title so that readers know that this is a protocol for a preregistered study.

2. Please provide a brief description of CBT-T in the Introduction. For example, what are the core modules that are included in the 10 sessions (versus 20 session CBT).

3. In the Introduction, page 4, the authors state that a change of >1.13 points on the EDE-Q-global separates rapid responders from gradual responders. Please provide more context about how this threshold was selected.

4. Since the study includes adolescents, it may be helpful to specify a BMI percentile threshold that would exclude young individuals from participation.

5. Past data have consistently shown that early change in the first four weeks of treatment predict positive treatment outcomes. This being said, the authors are assessing whether a patient is a rapid or gradual responder based on the data collected from the first three sessions, as the assessment time point is prior to session 4. I wonder if moving the assessment time point prior to session 5 (aka, one session later) would more closely correspond to past research?

6. I highly recommend that the authors also include race/ethnicity and ED diagnosis as additional demographic variables.

7. What do the authors mean when they say that recruitment materials will be distributed to "headspaces?"

8. How did the authors choose their remission criteria?

Reviewer **2**

Name **Kirschbaum-Lesch, Inken**

Affiliation **Ruhr University Bochum, LWL-University Hospital for Child and Adolescent Psychiatry, Psychotherapy and Psychosomatic Medicine**

Date **19-Feb-2025**

COI **None**

A randomozed open-label trial of ten session cognitive behavior therapy (CBT-T) for eating disorders: Does stratified augmented treatment lead to better outcomes?

This study protocol describes a randomised, open-label trial of ten sessions of cognitive behavioural therapy (CBT-T) compared with CBT-T augmented with therapy modules (CBT-TA). CBT-TA is primary intended to help gradual responders after three treatment sessions. Gradual responders should select one of 9 therapy modules with their therapist and add this tot he therapy. Measurements are taken before therapy (T1), in the fourth treatment session (T2), after treatment (T3) and at a 3-month (T4) and 6-month follow-up (T5).
It is a well written study with a interesting and important research question. As the participants in both groups receive therapy that has already been show to be effective in previous studies, they have a good benefit from participating in the study. The research questions were well derived and the study design is adequate to answer the research questions. Methods and outcomes are clearly defined. Only minor revisions should be made.

Following aspects should be revised:

Abstract:

- Please use past tense when reporting previous study results „recent works showed"

- Please add the information how ED psychopathology will be assessed.

- Please add age of participants.

Introduction:

- Please explain acute aufmentation (l. 79).

- How is remission defined (l. 114)

Introduction or Method:

- Are there reasons to include from the age of 15? Please explain why you decided to include participants from the age of 15.

Methods:

- P. 9, l.184: I find it confusing that there are 3-Follow up times are mentioned, although there are just 2 Follow-Up assessment and one Post-Treatment Assessment (T3). I would suggest to define T3 as post-treatment and not as Follow-Up.
- P. 11, l. 218: „If" instead of „if".
- P. 11, l. 239: Which eating behaviors? Please add an example of disordered eating behaviors as in the case of eating and shape concerns.
- P. 13 One item on therapy expectation could be interesting in the baseline assessment.

Material in the Supplement

- Is there also a consent form for custodians?
- The title of your study is written on the consent form and contains the question whether augmented therapy is better than CBT-T alone. If I, as participant, know that I will get something extra compared to the other group, which is also the hypothesis of the study, could this not distort the results? I think the participants should be blind to the hypothesis which therapy is expected to be better. You may need a cover story explaining that two therapies will be compared and it is not known which therapy is better. But findings of previous studies showed that both therapies are effective.

Reviewer 3

Name Preuss-van Viersen, Hanna

Affiliation University Medical Center of the Johannes Gutenberg University Mainz, Department of Child and Adolescent Psychiatry and Psychotherapy

Date 07-Mar-2025

COI None

The study protocol entitled "A randomized open-label trial of ten-session cognitive behaviour therapy (CBT-T) for eating disorders: Does stratified augmented treatment lead to better outcomes?" (Manuscript ID bmjopen-2025-099212) reports on a very interesting

study design of personalized psychotherapy in non-underweight eating disorders that uses stratified treatment for gradual responder (CBT-TA) with participant choice for the therapy modules augmented. The protocol is well written and addresses a relevant topic (personalization of treatment). I believe the manuscript makes a substantial contribution to the literature and is worthy of publication. However, I would like to point out some comments that might help to increase the quality of this protocol.

ABSTRACT

- Primary and secondary outcomes should be mentioned in the abstract.

- Ethics approval and trial registration number are missing. Please give further information and add informations.

BACKGROUND/INTRODUCTION

- The comparability of CBT-T for adults and adolescents must be described (see also my comment under methods). As far as I know, there is only evidence for adults for brief cognitive-behavioral therapy for eating disorder within RCTs, the study of Hart et al. (2024) has no control group.

- The authors write „We also hypothesise that remission will be higher in the former than latter group." Given the range of eating disorder diagnoses included, it is unclear what exactly is meant by remission and how remission is defined.

- What is meant with change in health service utilisation?

- ED psychopathology measured with the EDE-Q global score is not the same as "disordered eating" - However, the EDE-Q global score is described as the primary outcome in the methods.

- Overall, the section on the research objectives and hypotheses would benefit from being more concise and clearly formulated.

METHODS

- There is no parental information sheet and consent form for young people between the ages of 15 and 18. Please submit the parental consent.

- Please describe the procedure of the Delphi consensus across four different panels in more detail.

- It is not clear to me why adolescents and adults are treated equally. In my view, the treatment of a 15-year-old requires age-appropriate adaptation of CBT. I would be in favor of limiting it to one age range.

- If you include adolescents, a body mass index (BMI) < 18.5 as exclusion criteria is not appropriate.

- On which data is the cut-off of EDE-Q≥1.13 for early responders and EDE-Q<1.13 for gradual responders based on?

- How is change in health service utilisation measured?

- Just as a small side note: In such an elaborate and high-quality study, a clinical judgment (EDE interview) would actually make sense as the primary outcome.

DISCUSSION

A section for discussion is missing, at least the strengths and weaknesses of the study should be named in detail.

Some very minor points:

-P. 9 line 145 EDA-5 online clinical interview is given as a link https://interview.eda5.org/index.html?t=eyJxIjoiZWRhNSIsIm1ham9yIjoxLCJtaW5vciI6Im

147 N1cnJlbnQiLCJsYW5nIjoiZW4iLCJkZWJ1ZyI6ZmFsc2V9\,

VERSION 1 - AUTHOR RESPONSE

#	Lines	Reviewer 1 comments	Our response
**4**	**9**	Please modify the title so that readers know that this is a protocol for a preregistered study.	Please see our response to point # 1; additionally, we have included the descriptor “pre-registered” in the title.
**5**	**72-75**	Please provide a brief description of CBT-T in the Introduction. For example, what are the core modules that are included in the 10 sessions (versus 20 session CBT).	We now provide a brief overview of the five phases of CBT-T and a key difference from longer forms of therapy.
**6**	**82-87**	In the Introduction, page 4, the authors state that a change of >1.13 points on the EDE-Q- global separates rapid responders from gradual responders. Please provide more context about how this threshold was selected.	We have provided the original study that first identified this change, and that this was based on reliable change index.
**7**	**179**	Since the study includes adolescents, it may be helpful to specify a BMI percentile threshold that would exclude young individuals from participation.	We now provide the equivalent for an adolescent, BMI-for-age <5th percentile.
**8**	**79-80**<br>**82-87**	Past data have consistently shown that early change in the first four weeks of treatment predict positive treatment outcomes. This being said, the authors are assessing whether a	We thank the reviewer for pointing this out. We chose session 4 as this is the review session. The 2021 Chang et al meta-analysis (“Early response to psychological treatment for eating

		patient is a rapid or gradual responder based on the data collected from the first three sessions, as the assessment time point is prior to session 4. I wonder if moving the assessment time point prior to session 5 (aka, one session later) would more closely correspond to past research?	disorders: A systematic review and meta-analysis“) in Clinical Psychology Review included papers that defined early change as occurring anywhere from between the first 3 to 9 weeks or sessions, with the majority examining change over the first 4 weeks or sessions. We note that we have validated change occurring before session 4 in our clinic in a reference 14. We have made this information clearer in the introduction.
**9**	**299-230**	I highly recommend that the authors also include race/ethnicity and ED diagnosis as additional demographic variables.	We do ask “what is your cultural identity”? We note that this issue needs to be addressed differently in each country, and in Australia we are currently barred by an Indigenous ethics policy asking people explicitly to disclose that they are of Aboriginal or Torres Strait Islander origin ((unless the research is focused specifically on Indigenous people). We have added the question to our demographic description. We will also have an ED diagnosis as outlined in the eligibility criteria and this has been added, as has socioeconomic status which will be generated from postcode of residence using Australian SEIFA coding.
**10**	**326-327**	What do the authors mean when they say that recruitment materials will be distributed to “headspaces?”	This refers to our primary mental health services for youth, called ‘headspace”. We have added that descriptor into the manuscript.
**11**	**367-369**	How did the authors choose their remission criteria?	The remission criteria are fairly widely applied and accepted in eating disorders, informed by the work of Bardone-Cone which was referenced where we refer to remission.
**#**	**Lines**	**Reviewer 2 comments**	**Our response**
**12**	**24**<br><br>**32-34**<br><br>**29**	Abstract:- Please use past tense when reporting previous study results „recent works showed“<br><br>- Please add the information how ED psychopathology will be assessed.<br><br>- Please add age of participants.	Past tense is now used.<br><br>This detail has been added.<br><br>This has been added.
**13**	**99-101**	Introduction:- Please explain acute augmentation (l. 79).	We have added a definition of acute augmentation.

	**367-369**	- How is remission defined (l. 114)	Remission was defined later in the paper.
**14**	**175-177**	Introduction or Method:- Are there reasons to include from the age of 15? Please explain why you decided to include participants from the age of 15.	We have supplied a rationale in the Method section.
**15**	**212-214**<br><br>**248**<br><br>**270**	Methods:- P. 9, l.184: I find it confusing that there are 3-Follow up times are mentioned, although there are just 2 Follow-Up assessment and one Post-Treatment Assessment (T3). I would suggest to define T3 as post-treatment and not as Follow-Up.<br><br>- P. 11, l. 218: „If“ instead of „if“.<br><br>- P. 11, l. 239: Which eating behaviors? Please add an example of disordered eating behaviors as in the case of eating and shape concerns.<br><br>- P. 13 One item on therapy expectation could be interesting in the baseline assessment.	We have reworded this section for greater clarity.<br><br>This has been corrected.<br><br>The 5 behavioural items have been specified.<br><br>We did consider this but decided against it as the therapies are identical from baseline to session 4 for all participants.
**16**		Material in the Supplement<br><br>- Is there also a consent form for custodians?<br><br>- The title of your study is written on the consent form and contains the question whether augmented therapy is better than CBT-T alone. If I, as participant, know that I will get something extra compared to the other group, which is also the hypothesis of the study, could this not distort the results? I think the participants should be blind to the hypothesis which therapy is expected to be better. You may need a cover story explaining that two therapies will be compared and it is not known which therapy is better. But findings of previous studies showed that both therapies are effective.	 <br>The consent form has for custodians has now been included.<br><br>Ethics requires us to be as transparent as possible with participants, but we have modified the title, with permission, from “A randomized trial of ten-session cognitive behaviour therapy (CBT-T) for eating disorders: Does stratified augmented treatment lead to better outcomes? to “A randomized trial of ten-session cognitive behaviour therapy (CBT-T) for eating disorders”.
**#**	**Lines**	**Reviewer 3 comments**	**Our response**

**17**	**39-41**<br><br>**43-48**	ABSTRACT<br>- Primary and secondary outcomes should be mentioned in the abstract.<br>- Ethics approval and trial registration number are missing. Please give further information and add informations.	The primary and secondary outcomes are now included.<br><br>This information was included in the Abstract.
**18**	**161-162**<br><br>**367-369**<br><br>**140-141**<br><br>**136, 152, 206**<br><br>**132-141**	BACKGROUND/INTRODUCTION<br>- The comparability of CBT-T for adults and adolescents must be described (see also my comment under methods). As far as I know, there is only evidence for adults for brief cognitive-behavioral therapy for eating disorder within RCTs, the study of Hart et al. (2024) has no control group.<br>- The authors write „We also hypothesise that remission will be higher in the former than latter group.“ Given the range of eating disorder diagnoses included, it is unclear what exactly is meant by remission and how remission is defined.<br>- What is meant with change in health service utilisation?<br>- ED psychopathology measured with the EDE-Q global score is not the same as “disordered eating” - However, the EDE-Q global score is described as the primary outcome in the methods.<br>- Overall, the section on the research objectives and hypotheses would benefit from being more concise and clearly formulated.	We now make it clear that all the four studies published from our clinic (Flinders University Services for Eating Disorders) have included participants aged 15 years and above. We could not locate a study by Hart et al (2024) in our manuscript.<br><br>Remission was defined later in the manuscript, adopting the widely used transdiagnostic definition which is not specific to diagnostic status.<br><br>We now specify that we refer to primary, secondary and tertiary care.<br><br>Where “disordered eating” was present it has now been changed to “ED psychopathology”.<br><br>This section has been reworded, but we have retained wording specified by our statistician.
**19**	 <br><br><br>**118-120**	METHODS<br>- There is no parental information sheet and consent form for young people between the ages of 15 and 18. Please submit the parental consent.<br>- Please describe the procedure of the Delphi consensus across four different panels in more detail.<br>- It is not clear to me why adolescents and adults are treated equally. In my view, the treatment of a 15-year-old requires age-	The consent form has for custodians has now been included.<br><br>We have included a little more detail as requested.<br><br>See our response to points # 14 and 18.

		appropriate adaptation of CBT. I would be in favor of limiting it to one age range.<br>- If you include adolescents, a body mass index (BMI) < 18.5 as exclusion criteria is not appropriate.<br>- On which data is the cut-off of EDE-Q≥1.13 for early responders and EDE-Q<1.13 for gradual responders based on?<br>- How is change in health service utilisation measured?<br>- Just as a small side note: In such an elaborate and high-quality study, a clinical judgment (EDE interview) would actually make sense as the primary outcome.	See our response to point # 7.<br><br>See our response to point # 6.<br><br>See our response to point # 18.<br><br>Thank you for this suggestion; given our previous four studies have used the EDE-Q (Self-report) as the primary outcome, we believe it makes sense to retain it in this study for reasons of comparability. We also note that global ED psychopathology scores on the interview and self-report versions of the EDE are highly corelated (.84).
**20**	**51-60**	DISCUSSION<br>A section for discussion is missing, at least the strengths and weaknesses of the study should be named in detail.	We defer to Editor advice in light of our response to point # 3, given the revised strengths and weaknesses are now highlighted earlier in the manuscript.
**21**	**171**	Some very minor points:<br>-P. 9 line 145 EDA-5 online clinical interview is given as a link	We have now shortened this link which is the primary way to access the interview: https://interview.eda5.org/

VERSION 2 - REVIEW

Reviewer 3

Name Preuss-van Viersen, Hanna

Affiliation University Medical Center of the Johannes Gutenberg University Mainz, Department of Child and Adolescent Psychiatry and Psychotherapy

Date 04-Apr-2025

COI

Thank you very much for the revisions and the detailed responses to my comments. The quality of the study protocol has improved significantly, open questions have been clarified. I also appreciate the revisions to the highlights. However, in my opinion, the critical evaluation is still not sufficiently addressed. I would highly prefer it if a small section discussing the strengths and limitations in the study proctocol could be added.
